# Analysing the intra and interregional components of spatial accessibility gravity model to capture the level of equity in the distribution of hospital services in Italy: do they influence patient mobility?

**DOI:** 10.1186/s12913-024-11411-3

**Published:** 2024-08-23

**Authors:** Fabrizio Pecoraro, Marco Cellini, Daniela Luzi, Fabrizio Clemente

**Affiliations:** 1 Institute for Research on Population and Social Policies, National Research Council (IRPPS-CNR), via Palestro 32 – 00185, Rome, Italy; 2 Institute of Crystallography, National Research Council (IC-CNR), Strada Provinciale 35d, 9 – 00010 – Montelibretti, Rome, Italy

**Keywords:** Spatial accessibility, Passive mobility, Hip replacement procedure, Universal healthcare coverage, Health system quality measurement

## Abstract

**Background:**

An equal distribution of hospital structures represents an important factor to achieve Universal Health Coverage. Generally, the most diffused approach to measure the potential availability to healthcare is the provider-to-population ratio based on the number of beds or professionals. However, this approach considers only the availability of resources provided at regional or local level ignoring the spatial accessibility of interregional facilities that are particularly accessed by patients living at the borders. Aim of this study is to outline the distribution of the intra and interregional services in Italy to capture the level of equity across the country. Moreover, it explores the impact of the accessibility to these resources on interregional patient’s mobility to receive care.

**Methods:**

To compute spatial accessibility, we propose an alternative approach that applies the enhanced two-step floating catchment area (ESFCA) to capture the level of attraction of intra and interregional hospitals to a given population. Moreover, the adoption of process and outcome indices captured to what extent the quality of structures influenced patients in choosing services located inside or outside their region of residence.

**Results:**

The study confirms that there is an unequal distribution of high-quality resources at regional and national level with a high level of inequality in the availability and accessibility of quality resources between the north and south part of Italy. This is particularly true considering the accessibility of intraregional resources in the southern part of the country that clearly influences patient choice and contribute to a significant cross border passive mobility to northern regions. This is confirmed by an econometric model that showed a significant effect of spatial accessibility with the propensity of patients of travel from the region of residence to receive care.

**Conclusions:**

The analysis of intra and interregional components of spatial accessibility may contribute to identify to what extent patients are willing to travel outside their region of residence to access to care services. Moreover, it can contribute to gain a deeper understanding of the allocation of health resources providing input for policy makers on the basis of the principles of service accessibility in order to contain patient mobility.

## Background

As stated by the World Health Organization (WHO) [1] Universal Health Coverage (UHC) means that all people have access to the full range of quality health services they need, when and where they need them, without financial hardship. It covers the full continuum of essential health services, from health promotion to prevention, treatment, rehabilitation and palliative care [1]. The provision of care services should give equal opportunity regardless of personal and territorial characteristics, such as gender, age, race, ethnicity, socioeconomic status [2, 3]. Access to services and providers is considered a central aspect to assess the performance of health systems with a prominent role not only for national and international bodies, but also in the health policy literature [4, 5]. It is generally associated with the concept of equity as it summarizes the opportunity of patients and communities to use appropriate services taking into account their needs [4, 6]. Different frameworks have been proposed in the literature to frame this multi-faced concept [4, 5, 7]. Among them, Levesque et al. [4], proposed a two-layer framework based on five dimensions (i.e., Approachability; Acceptability; Availability; Affordability and Appropriateness) and as many persons’ abilities (Perceiving; Searching; Reaching; Paying; Engaging). From our perspective, this paper concentrates the attention on geographic accessibility and the relevant ability to reach the service that is considered a concern of growing importance to policy makers as spatial barriers may contribute to lower health care utilization and outcomes [8].

Achieving UHC is an emerging priority of health systems worldwide, and one of the 9 outcome targets listed in the third Sustainable Development Goal (SDG), regarding “Good Health and Well-being” [9]. The level of UHC is computed adopting a composite indicator comprising 14 tracer measures organized by four broad categories [10]: reproductive, maternal, new-born and child health; infectious diseases; noncommunicable diseases; service capacity and access. Within the latter category three indicators are captured to assess the level of service coverage: 1) hospital beds density; 2) health professionals per capita; 3) international Health Regulations (IHR) index which measures the capacity of a country for early warning, risk reduction and management of health risks. Thus, the capacity of hospital structures in terms of beds and professionals represents an important factor to achieve UHC. Generally, the most diffused approach to measure potential availability to healthcare is the provider-to-population ratio computed as the number of beds or professionals per population, as also adopted in the SDG 3.8 indicators 13 and 14 [10]. However, this approach considers only the availability of adequate health care resources and does not take into account a fundamental aspect of universal care: the accessibility in terms of travel distance [11]. Availability and accessibility are commonly merged under the term spatial accessibility [12–15]. Moreover, the provider-to-population indicator considers only the availability of intraregional resources, ignoring the spatial accessibility of interregional facilities that are particularly accessed by patients living at the borders not only at regional level [16, 17] but also across countries [18]. Although the majority of national and international authorities still rely on these provider-to-population indicators, different methodologies have been proposed in the literature to overcome these limits [17, 19–21]. They are mainly based on the gravity-based focusing in the provision of healthcare services at different geographical scales (e.g., urban, regional, or national). Among them, recently, a wider attention has been paid to the enhanced two-step floating catchment area (E2SFCA) method that has been extended and modified to fit with the purpose of specific case studies [22–25].

Travelling across a country for accessing care services is implicitly encouraged in those national healthcare systems (e.g., Italy, Denmark, England, the Netherlands, Sweden) where the introduction of policies allows patients to freely choose any public or private provider in the country. From a patient perspective, the opportunity of moving beyond borders may have two opposite implications. On the one hand it can improve the UHC since patients may decide where to be treated on the basis of their needs as well as considering accessibility and quality of services. On the other hand, this opportunity may be hindered by different socio-economic and demographic individual factors [26, 27], such as income, propensity to travel, education level, age, pathology complexity. These factors can represent barriers influencing patient choice and limiting the propensity of patients to travel across the country to be treated in high-quality structures [28]. From a health system perspective, the introduction of these free-to-choice policies has created financial incentives for providers to compete among them and have also led to improvements in the quality of care [29–31]. Moreover, in Italy, treating not resident patients implies economic compensation procedures between the patient’s region of residence and the one that provides the service [32]. This complex migratory phenomenon where patients benefit from the healthcare services outside their region of residence is called passive mobility [17, 33]. In Italy, mitigating this phenomenon was one of the main actions planned within the Health Pact 2019–2021 [34] as it is considered as a proxy for the quality and availability of hospital services [30, 35, 36] highlighting significant socio-economic disparities at regional level [28, 37]. This is particularly evident in Italy where, compared to other European countries, patients tend more frequently to travel long distances to access to care [38] especially for elective treatments [29]. Patient mobility has been widely studied to capture factors that may influence the patients’ choice, including social, demographic and economic status [39], quality and complexity of regional services [29] as well as structural components related to personnel, technologies and equipment available [29].

Within this context, the aim of this study is twofold. Firstly, it provides an analysis of the intra and interregional components of the hospital spatial accessibility outlining a snapshot of the distribution of services to capture the level of equity across the country [12, 17]. Secondly, it explores the impact of the accessibility to these resources on patient’s mobility [17]. In this way, this study analysed the role of intra and interregional components combining the potential spatial accessibility to care resources with utilization spatial measures at local level (i.e., passive mobility). This analysis may provide an input for policy makers to capture to what extent the capacity and distribution of hospitals and services may affect patient’s flows at regional level. To accomplish this aim an extended approach to capture the inter-regional component of spatial accessibility is proposed in the next paragraph starting from the methodologies published in the literature and based on the capacity and quality of healthcare facilities. The service accessibility indicators defined adopting this methodology are subsequently analysed to capture how facilities and services are distributed over the Italian territory and to what extent they impact on patient mobility.

From a clinical perspective, although it is widely recognized that UHC depends on a strong primary health-care system [40] surgery and surgical health services play an essential role in the provision of equitable and feasible services [41, 42]. For this reason, this study focuses the attention on hip replacement procedure, a high specialization procedure where patients are generally prepared to travel long distances [43] and around 20% of them are treated outside their region of residence [44]. Moreover, from an organizational perspective, this elective treatment does not require a long pre- and post-hospitalization period, rehabilitation can be carried out in other structures closest to the place of residence, it is not an emergency procedure so the patients can, generally, program the intervention with the support of the primary care and the family taking into account the waiting times and without rushing.

## Materials and methods

### Service accessibility indicators

Among the different methods proposed in the literature the two-step floating catchment area (2SFCA) [45] and its enhanced version (E2SFCA) [46] have been widely adopted to assess spatial accessibility [47]. The latter one is based on the gravity model [45] and assumes that the level of attraction of each facility on a population is directly related with its capacity and inversely associated with the distance between them [48]. This methodology firstly introduced a decay weight that proportionally assesses the probability that a patient accesses a given hospital considering the distance between the patient’s residence and the place of care based on a catchment area (i.e., maximum distance patients are willing to travel to access to a service). This methodology entails two steps [46]. In the first one the supply-to-demand ratio (*R*_*j*_) is computed for each facility *j* by dividing the relevant capacity variable (*n*_*j*_) with the potential demand (*P*_*j*_), as reported in Eq. 1.

1 $${R}_{j}=\frac{{n}_{j}}{{P}_{j}}=\frac{{n}_{j}}{{\sum }_{i}{P}_{i}*{w}_{ij}}$$

Where *P*_*j*_ represents the distance-weighted sum of the population falling within a specified threshold distance (*d*) of facility *j*, *P*_*i*_ is the population resident in the municipality *i* and *w*_*ij*_ is the weight assigned to distance *d*_*ij*_ (i.e., distance between the municipality *i* the structure *j*) based on a distance decay function (see Eq. 2). Note that to avoid possible bias due to an inhomogeneous distribution of demographic characteristics the reference population of each municipality is limited to residents aged 55 and over, considering that, in Italy, this age range covers about 95% of the proportion of patients (i.e., incidence) who underwent hip replace surgery, with no significant differences between sexes. Unfortunately, due to the lack of data at municipality level, we cannot consider other aspects such as pathologies or frailness. The choice of the decay function is generally based not only on the dimension of the catchment area, but also on the type and velocity of decay (i.e., to what extent each added travel minute is felt by the patient), such as Gaussian [49], exponential [50], inverse power [51] and kernel density [52]. In particular, in this paper, as well as in our previous studies [17, 53], we adopted the Gaussian function (Eq. 2) where *d* represents the distance factor (i.e., threshold) and *d*_*ij*_ specifies the time taken by a car to cover the distance between the centroids of the municipalities *i* and *j*. This is preferred in particular in gravity models such as the E2SFCA as it considers the accessibility attenuation rate to be slower both in the near and far stages than in the middle ones [23]. It is important to highlight that, the issue of choosing the catchment area and the distance decay function [14, 54] as well as the transportation modes included in the model [55, 56] is well known and their selection have a large effect on the resulting accessibility indices as they govern the conversion of a measured distance in minutes to weight value. However, as observed data that can be used to estimate a realistic distance-weight relationship is not available, we choose 120 min as the catchment area factor of the Gaussian decay function, which is in line with the current literature [13, 57, 58].

2 $${w}_{ij}={e}^{-\frac{{d}_{ij}^{2}}{0.2*{d}^{2}}}$$

In the second step of the methodology the distance-weighted sum of the supply-to-demand ratios is computed for each population unit *i* on the basis of Eq. 3.

3 $${A}_{i}={\sum }_{j}{R}_{j}*{w}_{ij}={\sum }_{j}\frac{{n}_{j}}{{\sum }_{i}{P}_{i}*{w}_{ij}}*{w}_{ij}$$

As previously mentioned, generally, ${n}_{j}$ represents a structural variable such as the number of hospital beds or physicians, while only a limited number of studies have focused the attention on the level of accessibility considering process indicators [59, 60] (e.g., number of visits) and none of the them to our knowledge have considered outcome measures to compute this equation. In this paper, our intent is to identify which are the main factors influencing patient’s choice in travelling outside their region of residence to be treated. For this reason, the accessibility index has been computed considering two perspectives: 1) the number of interventions *(int)* performed by a hospital as a critical factor that may drive patient demand as it is well known that performing a high number of interventions may lead to a high quality of service [61, 62]; 2) the readmission rate within 30 days after discharge (*ret*_*30*_) as a proxy for the quality and efficacy of surgical procedures as reported by the Italian National Outcome Programme (PNE) [44, 63] as well as by the literature [64, 65]. These two variables represent an alternative perspective in comparison to structural ones as they are not strictly related with the capacity of the hospital, but on the quality of care that may represent an important factor to be considered by patients when choosing a hospital. Moreover, it is difficult to capture to what extent structural components are dedicated to a specific service. For instance, looking at the hip replacement surgery, it is variable and unknown what is the portion of beds available in the orthopaedics wards specifically devoted to this procedure.

These quality indicators are collected by the PNE considering a time period of one calendar year and adopted to assess and compare the quality of hospital structures. A summary description of the outcome indicator (*ret*_*30*_) is reported in Table 1 to highlight its definition, numerator and denominator as well as relevant statistical and methodological notes. *ret*_*30*_ and *int* have been adopted to identify the level of adherence to quality standards as established by the Italian regulation regarding the definition of hospital standards [44, 63, 66]. In particular, for the purpose of our analysis a structure that performed less than 73 interventions in given year (i.e., PNE set a threshold of 80 interventions with a tolerance of 10%) is not considered attractive to patients and its quality level indicator (*ql*) is set to 0. On the contrary, if this intervention threshold is guaranteed, the quality level of the structure is inversely proportional to *ret*_*30*_ as summarized in Table 1.

**Table 1:** Methodological and statistical characteristics of the outcome indicator

Indicator	Readmission 30 days after hip prosthesis surgery *(ret*_*30*_*)*
Definition	By hospital structure or area of residence: proportion of hospitalizations with readmission within 30 days from the date of hip replacement surgery
Numerator	Number of hospitalizations with readmission within 30 days from the date of discharge of hospitalization for hip prosthesis
Denominator	Number of hospitalizations with hip prosthesis surgery
Statistical methodology	The comparative assessment takes into account the lack of territorial homogeneities existing in the populations studied, due to gender, age and a set of comorbidities affecting the patient. These characteristics have been adopted to adjust the return ratio that allows to study the differences between structures performances removing possible confounding effect of the uneven distribution of patient characteristics
Quality level	If the number of interventions (*int*) performed in the reference year is higher than 72 (*int* > 72): • *ret*_*30*_ ≤ 3, *ql* = 1 • 3 < *ret*_*30*_ ≤ 4.5, *ql* = 0.8 • 4.5 < *ret*_*30*_ ≤ 6, *ql* = 0.6 • 6 < *ret*_*30*_ ≤ 7.5, *ql* = 0.4 • 7.5 < *ret*_*30*_ ≤ 9, *ql* = 0.2 • *ret*_*30*_ > 9, *ql* = 0 If the number of interventions performed in the reference year is lower than or equal to 72 (*int* ≤ 72) the quality level is automatically set to 0 * Thresholds and classifications are set by the Italian regulation and available at the PNE website [44, 63, 66]
Note	The volume of hospitalizations for surgical operations is calculated on an annual basis, referring to the year of discharge from the hospitalization

The number of interventions (*int*) and the quality level (*ql*) have been subsequently adopted to qualify each hospital structure on the basis of Eq. 3 (see Eqs. 4 and 5).

4 $${R}_{j}^{int}=\frac{{int}_{j}}{{\sum }_{i}{P}_{i}*{w}_{ij}}$$

5 $${R}_{j}^{ql}=\frac{{ql}_{j}}{{\sum }_{i}{P}_{i}*{w}_{ij}}$$

**Fig. 1:**
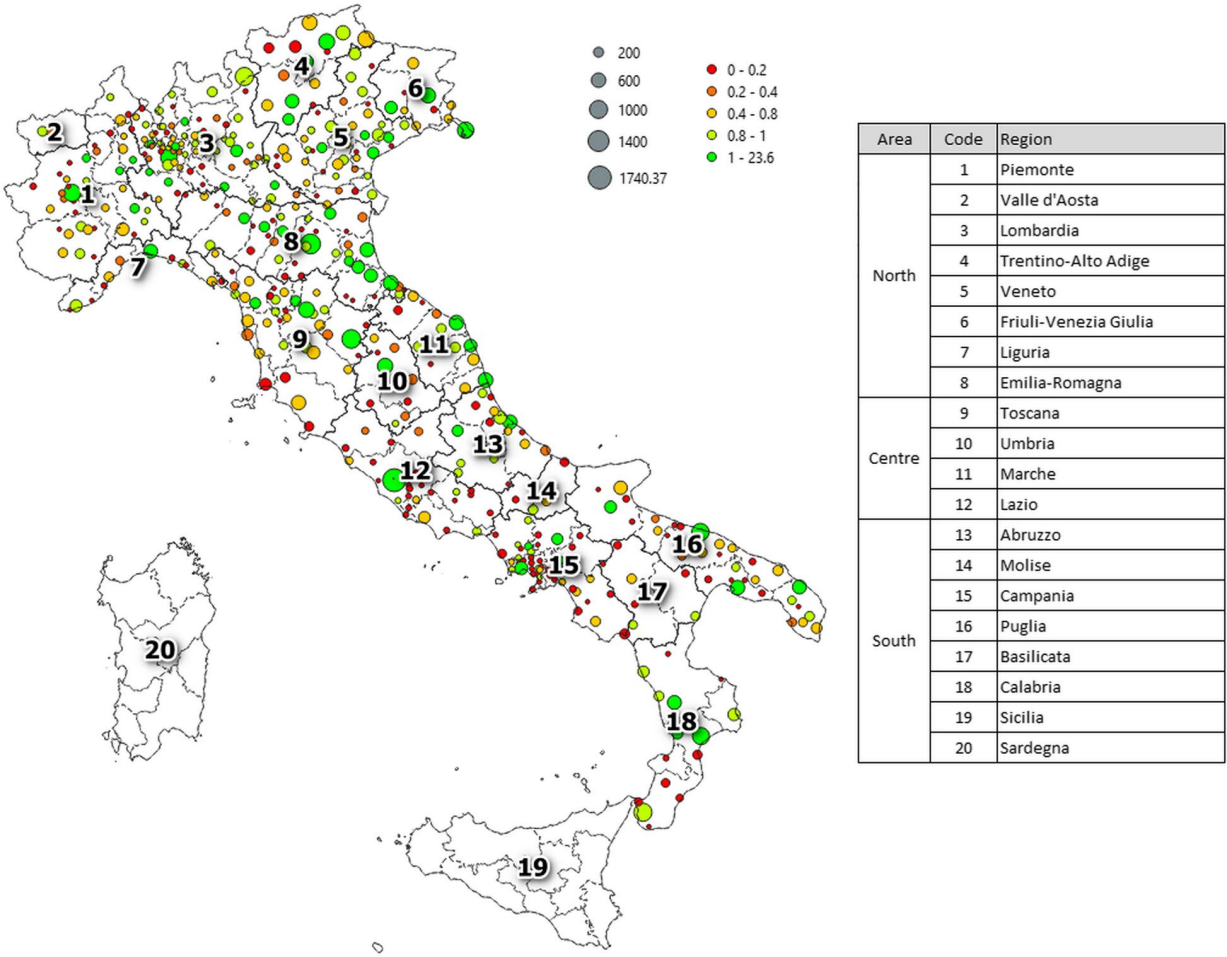
Distribution of the hospital structures. Legend: Size of circels is proportional to the number of interventions (${\overline{\text{int}} }_{\text{j}}$), while colour of circles is related with the quality level (${\overline{\text{ql}} }_{\text{j}}$). Hospitals are aggregated at municipality level. Date refers to the year 2021

Figure 1 shows the distribution of hospital structures over the Italian territory for the year 2021 highlighting the number of interventions performed (i.e., size of the centroid proportional to ${\overline{int} }_{j}$) and the quality level (i.e., colour of the centroid related with ${\overline{ql} }_{j}$). Note that, as the distance between municipalities are computed considering travel times by private car, islands cannot be included in the analysis as residents cannot access to interregional facilities. For this reason, hospitals located in Sardinia and Sicily as well as in the other islands are not shown in Fig. 1.

Starting from Eqs. 4, 5, it is possible to compute the accessibility indices based on each of the two measures, as shown in Eqs. 6, 7.

6 $${I}_{i}={\sum }_{j}{R}_{j}^{int}*{w}_{ij}$$

7 $${Q}_{i}={\sum }_{j}{R}_{j}^{ql}*{w}_{ij}$$

These two indicators (${I}_{i}$ and ${Q}_{i}$) provide an overall picture of the accessibility level for each municipality. However, they do not distinguish between hospitals placed inside or outside the patient’s region of residence. Thus, the following step of the methodology is to determine the contribution of the intraregional and interregional structures to the whole accessibility. This decomposition is particularly important for assessing differences across regions in countries like Italy where a decentralized organizational structure is in place and where regions are responsible for organizing and delivering health care through their belonging local health authorities responsible for delivering public health, community health services as well as primary and secondary care. This can help us to capture to what extent these components may impact on patient mobility. In particular, Eq. 8 reports how the intraregional component is measured taking into account the number of interventions (*int*).

8 $${I}_{i}^{INTRA}={\sum }_{j\in \{Reg\left(i\right)=Reg\left(j\right)\}}{R}_{j}^{int}*{w}_{ij}$$

Substantially, the intraregional component of this indicator is computed considering only hospitals that belong to the same region of residence of the patient.

Similarly, it is possible to apply the same equation to compute the interregional component of each index by considering hospitals *j* that are located outside the patient’s region of residence (i.e., where $j\in \{Reg\left(i\right)\ne Reg\left(j\right)\}$).

Finally, on the basis of the gravitational model a composite indicator can be computed by subtracting the intraregional with the interregional component of the same index so to capture to what extent the level of attraction differs between these two components (see Eq. 9).

9 $${I}_{i}^{G}={I}_{i}^{INTRA}-{I}_{i}^{INTER}$$

Final step of the methodology is to aggregate these indicators at province level calculating the weighted average of the single municipality with the reference resident population. For a given province *p* this is done by applying the Eq. 10.

10 $${I}_{p}^{G}=\frac{{\sum }_{i\in \{Prov\left(i\right)=p\}}{I}_{i}^{G} * {P}_{i}}{{\sum }_{i\in \{Prov\left(i\right)=p\}}{P}_{i}}$$

### Patient mobility

Passive mobility index (*M*) is defined has the percentage of patients that reside in a given place (i.e., province) that access to structures located outside their region of residence. It is computed as the ratio between the number of patients treated outside their region of residence (${paz}_{p}^{INTER}$) and the total number of patients treated (${paz}_{p}={paz}_{p}^{INTRA}+{paz}_{p}^{INTER}$) (see Eq. 11).

11 $${M}_{p}=\frac{{paz}_{p}^{INTER}}{{paz}_{p}^{INTRA}+{paz}_{p}^{INTER}}=\frac{{paz}_{p}^{INTER}}{{paz}_{p}}$$

### Data source

Data was obtained from the PNE website [42], an observatory that yearly monitors the effectiveness, appropriateness and safety of health interventions, aimed at improving the quality of care of the National Health Service. Based on hospital discharge records collected for both public and private hospitals, the PNE analyses 170 process and outcome indicators referred to 12 nosological scopes, publishing them at the end of the following year (i.e., data of 2022 have been published in November 2023). In this paper, the attention is focused on the hip replacement surgery procedure. Data on number of interventions (*int*) and readmissions within 30 days from the date of surgery (*ret*_*30*_) refers to the three-year period 2020–2022. This provides a picture of during and post COVID-19 pandemics to capture its impact on the mobility across regions, considering the travel restrictions adopted by the Italian and the regional governments as well as the cancellation or postponement of this type of interventions due to the use of hospitals to treat patients infected by COVID or due to organizational reasons. Considering the number of interventions in this three-years period there was a counter-tendency with respect to the years before COVID when the hip replacement procedures constantly raised about 2/3% per year from 105 thousands procedures (in 2015) to 116 thousands (in 2019). In 2020, they dropped to 97 thousands, but the two years after they have returned to pre-COVID numbers with an interesting peak of interventions of 125 thousands in 2022 [42]. Moreover, PNE provides for each Italian province the number of patients who underwent surgery inside or outside their region of residence. This data is adopted to capture the interregional passive mobility at province level.

To compute the distance between municipalities we adopted origin–destination matrix published by the Italian Institute of Statistics (ISTAT) that provides travel times in minutes between Italian municipalities by private car [67]. As previously explained islands are be included in the analysis as residents cannot access to interregional facilities by private car.

Additional variables related to socio-economic and territorial factors (Table 2) have been also collected and included in the analysis to assess their strength on patient mobility as well as to remove possible biases due to their distribution on the territory. They have been collected from the ISTAT [68], EUROSTAT [69] and the Italian Ministry of Health [70] websites, based on the relevant literature [28, 37].

**Table 2:** Characteristics of the socio-economic and territorial factors included in the extended econometric model

**Indicator**	**Description**	**Source**	**Granularity**	**Access date**
Position	Classification of the territorial within the first-level NUTS of the European Union: north, centre and south. North east and west were merged in the north class, while Islands were excluded from the model	EUROSTAT	Municipality	December 2023
Income	Average gross income of natural persons	ISTAT	Municipality	December 2023
Education level	Percentage of residence with at least a high school diploma	ISTAT	Municipality	December 2023
Waiting times	Number of days (average) for access to hip prosthesis surgery	Ministry of health	Region	December 2023
Health expenditure	Current health expenditure per capita (general)	ISTAT	Region	December 2023
Specialists	Number of specialists per population (10.000 inhabitants) (all specialties)	ISTAT	Region	December 2023
Satisfaction	Level of patient satisfaction due to the last hospital admission	ISTAT	Region	December 2023

### Analytic approaches

Giver the nature of the data, the effect of the accessibility indices on passive mobility has been assessed using a panel analysis with a fixed effect model, as suggested by the results of the Hausman test [71]. Subsequently, we identified and excluded outliers from the analysis using the Cook’s distance [72]. Finally, robust standard errors have been calculated since the Breusch–Pagan test [73] revealed the presence of heteroscedasticity. Note that, to avoid possible biases due to the different scales in which variable are expressed and to facilitate results’ interpretation, all the variables have been standardized between 0 and 1. Moreover, to capture the effect of the accessibility indices as well as of socio-economic and territorial factors, in the econometric model these variables refer to the previous year with respect to the mobility data. Thus, independent variables refer to the time period 2019–2021 while passive mobility to the period 2020–2022.

Considering that the variable year is not included in the panel analysis, a one-way analysis of variance (ANOVA) followed by a set of paired student tests have been performed to test whether passive mobility differs between years. The result of this analysis highlighted a statistically significant difference between the three years under investigation with the *p*-values of both ANOVA and student tests lower than 0.001 and an average (standard deviation) passive mobility of 19.1% (12.1%) in 2020, 21.5% (13.8%) in 2021 and 22.9% (13.9%) in 2022.

## Results

### Descriptive analysis

**Fig. 2:**
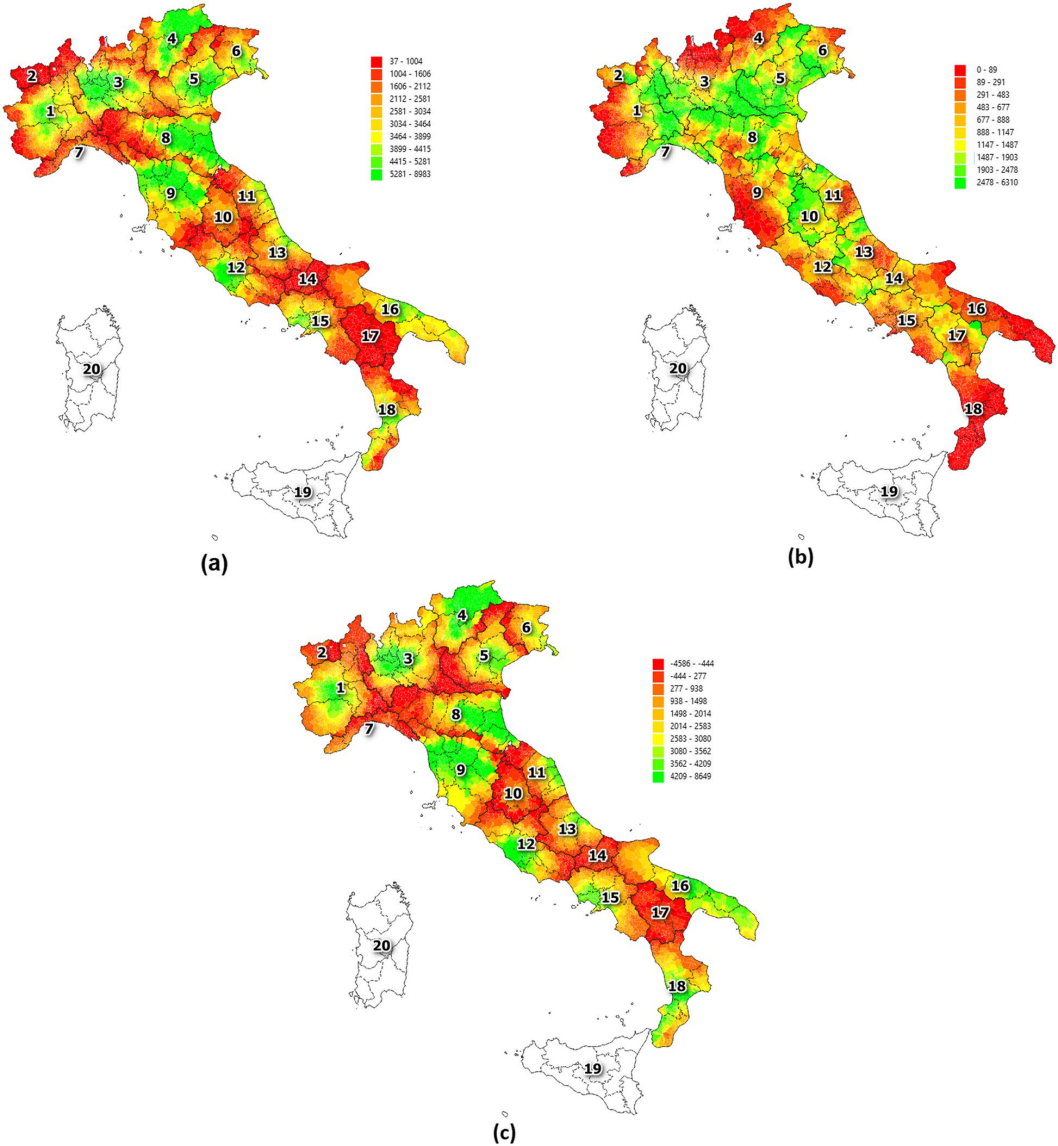
Accessibility index of interventions. Legend: Accessibility index considering the distribution of the number of Interventions (int) at municipality level for intra (**a**) interregional (**b**) components as well as gravity model (**c**). Reference year: 2021

**Fig. 3:**
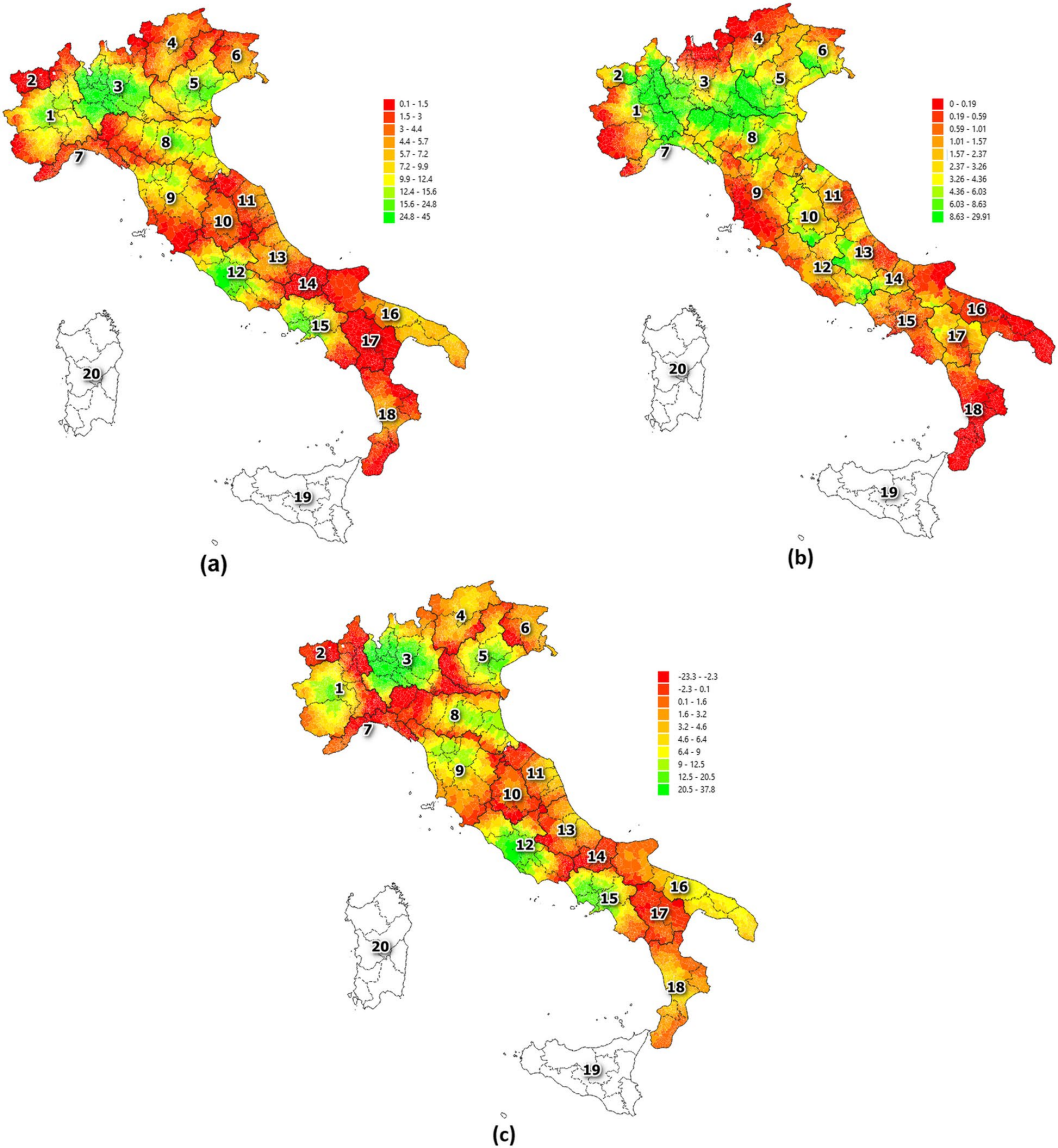
Accessibility index considering of quality level. Legend: Accessibility index considering the distribution of the quality level (ql) at municipality level for intra (**a**) interregional (**b**) components as well as gravity model (**c**). Reference year: 2021

Figures 2 and 3 show the distribution of the two accessibility indices (respectively ${I}_{i}$ and ${Q}_{i}$) over the territory, taking into account their intra (i.e., ${I}_{i}^{INTRA}$ and ${Q}_{i}^{INTRA}$) and interregional (i.e., ${I}_{i}^{INTER}$ and ${Q}_{i}^{INTER}$) components as well as the gravity model (${I}_{i}^{G}$ and ${Q}_{i}^{G}$) for the year 2021. Average values of the two indicators are reported in Tables 3 and 4 distributed at regional level. Raw data with the lowest level of granularity as well as data aggregated at province level are available from the Zenodo platform [74]. Data refers to hospitalization for hip replacement surgery.

**Table 3:** Accessibility Index at regional level considering the number of Interventions (int)

**Region**	**2019**			**2020**			**2021**		
	**intra**	**inter**	**gravity**	**intra**	**inter**	**gravity**	**intra**	**inter**	**gravity**
Abruzzo	2966.8	1508.4	1458.4	2599.4	1334.8	1264.6	3030.2	1577.2	1452.9
Basilicata	629.1	1154.6	-525.5	508.3	1046.3	-538.0	600.6	1227.8	-627.2
Calabria	2922.3	86.0	2836.3	2538.2	67.4	2470.8	2996.0	93.6	2902.4
Campania	3545.8	383.4	3162.4	3080.2	363.1	2717.1	3802.2	424.0	3378.2
Emilia-Romagna	4947.8	1955.2	2992.5	4230.8	1591.8	2639.1	4991.7	1842.1	3149.6
Friuli Venezia Giulia	3674.0	1424.5	2249.5	3326.7	1267.1	2059.6	3652.8	1464.8	2188.0
Lazio	4317.4	741.5	3575.9	3856.3	610.4	3245.8	4532.0	695.8	3836.2
Liguria	2516.9	1602.0	914.9	1756.4	1296.3	460.1	1940.5	1656.3	284.2
Lombardia	5599.9	1800.2	3799.7	3975.8	1506.9	2468.9	5207.6	1782.7	3424.9
Marche	3082.2	1163.8	1918.4	2868.7	1031.9	1836.8	3063.7	1163.9	1899.8
Molise	803.9	1071.0	-267.2	626.5	963.0	-336.5	784.3	1111.4	-327.1
Piemonte	3804.1	1514.6	2289.5	3055.4	1074.7	1980.7	3849.8	1380.0	2469.9
Puglia	3325.5	200.1	3125.4	3116.3	165.0	2951.3	3402.1	183.1	3219.0
Toscana	5579.5	897.3	4682.2	4864.5	745.1	4119.4	5360.9	860.6	4500.3
Trentino-Alto Adige	5324.3	903.0	4421.3	4069.5	727.2	3342.3	5696.5	840.7	4855.8
Umbria	2356.9	2358.3	-1.4	2108.9	2143.2	-34.3	2126.7	2156.1	-29.4
Valle d’Aosta	749.3	1591.2	-841.8	570.6	1256.1	-685.5	822.2	1539.3	-717.1
Veneto	4464.3	2293.6	2170.8	3949.5	1917.4	2032.0	4287.2	2411.8	1875.4
Italy	4244.3	1287.5	2956.8	3515.2	1066.0	2449.1	4185.3	1269.6	2915.7

**Table 4:** Accessibility index at regional level considering the quality level (ql)

**Region**	**2019**			**2020**			**2021**		
	**intra**	**inter**	**gravity**	**intra**	**inter**	**gravity**	**intra**	**inter**	**gravity**
Abruzzo	4.93	2.69	2.24	3.66	2.57	1.10	4.64	2.76	1.88
Basilicata	0.74	2.39	-1.65	0.67	2.03	-1.36	0.76	2.37	-1.61
Calabria	2.03	0.10	1.92	2.48	0.03	2.44	3.13	0.17	2.96
Campania	13.47	0.79	12.69	12.69	0.68	12.01	15.38	0.82	14.57
Emilia-Romagna	12.02	7.06	4.96	9.37	5.69	3.68	11.56	6.25	5.31
Friuli Venezia Giulia	6.00	4.09	1.91	4.83	3.48	1.34	5.42	4.00	1.42
Lazio	23.14	1.85	21.29	16.26	1.57	14.69	19.56	2.02	17.55
Liguria	3.88	6.05	-2.17	3.19	4.36	-1.17	3.68	5.77	-2.09
Lombardia	36.38	6.60	29.78	25.17	4.36	20.81	31.62	6.05	25.57
Marche	3.86	1.89	1.97	3.96	1.73	2.23	3.89	1.93	1.96
Molise	1.59	2.45	-0.87	1.06	2.38	-1.33	0.93	2.78	-1.84
Piemonte	15.22	7.97	7.25	10.84	5.74	5.10	12.22	7.03	5.19
Puglia	4.95	0.35	4.60	5.26	0.35	4.91	5.77	0.26	5.52
Toscana	7.91	1.95	5.95	8.27	1.40	6.87	9.45	1.80	7.65
Trentino-Alto Adige	3.94	2.57	1.37	4.26	2.17	2.09	5.69	2.55	3.14
Umbria	3.71	4.89	-1.18	3.85	4.01	-0.16	3.80	4.18	-0.39
Valle d’Aosta	1.22	7.96	-6.74	1.76	5.75	-4.00	1.22	6.57	-5.35
Veneto	14.43	6.58	7.85	12.71	5.32	7.39	12.78	6.20	6.58
Italy	15.81	4.20	11.60	12.16	3.16	8.99	14.49	3.90	10.59

Low accessibility in terms of both intra and interregional facilities can be found in the south of the country considering both the number of interventions and the quality level. Exceptions can be noted in the Puglia and Campania due to the presence of structures placed in and around their biggest cities (respectively Bari and Naples). Looking at the central regions, a low level of accessibility can be found in particular considering the intraregional component of both indicators. High level of accessibility is present in the Lazio region and in Tuscany, where hospitals performed a high volume of surgery procedures, in particular those placed in and around the cities of Rome and Florence. A particular case is Umbria, where the low level of intraregional accessibility is compensated by the high level of the interregional one. This is also due to the highway infrastructures that connect this region with hospitals located in Rome, Florence and the major cities of Emilia Romagna. The majority of the territory covered by a high level of accessibility is placed in the northern part of the country considering both the intra and interregional components of both indices. Of course, the geographic distribution of hospitals and the availability of high-speed network have an important impact along with the conformity of the territory, as highlighted by the mountain regions (Trentino-Alto Adige, Liguria and Val d’Aosta). The inequality in the accessibility indices at regional level found from the north to the south of the country is mainly due to the concentration of hospitals in high populated zones and in particular in big cities such as Rome (Lazio), Milan (Lombardia), Naples (Campania). The main impact of this concentrated distribution of hospitals mainly results in leaving rural areas not served or far away from these surgical and curative services. On the contrary, other regions such as Basilicata and Molise, despite their low level of both accessibilities, report a fairly distribution of structures localizing them also at their borders resulting in a high level of inflow mobility rate from other regions [75].

Analysing the gravity model component (see Figs. 2c and 3c), it emerges that five regions (Basilicata, Liguria, Molise, Umbria, Val d’Aosta) reported a negative value for both number of interventions and quality level indicators highlighting that the accessibility to interregional structures exceeds the intraregional ones. Toscana and Trentino Alto Adige represent the two regions with the highest capacity but, as a counterpart, with a relatively low-quality level. This is mainly due a massive number of interventions performed by intraregional hospital which are however distributed in several hospitals with a high risk of return. The three already mentioned regions (Lombardia, Lazio, Campania) which have very large cities (Milan, Rome, Naples) reported high values for both capacity and quality level indicators suggesting that in these territories the high number of interventions are concentrated in a low number of hospitals that also provide high quality level services.

**Fig. 4:**
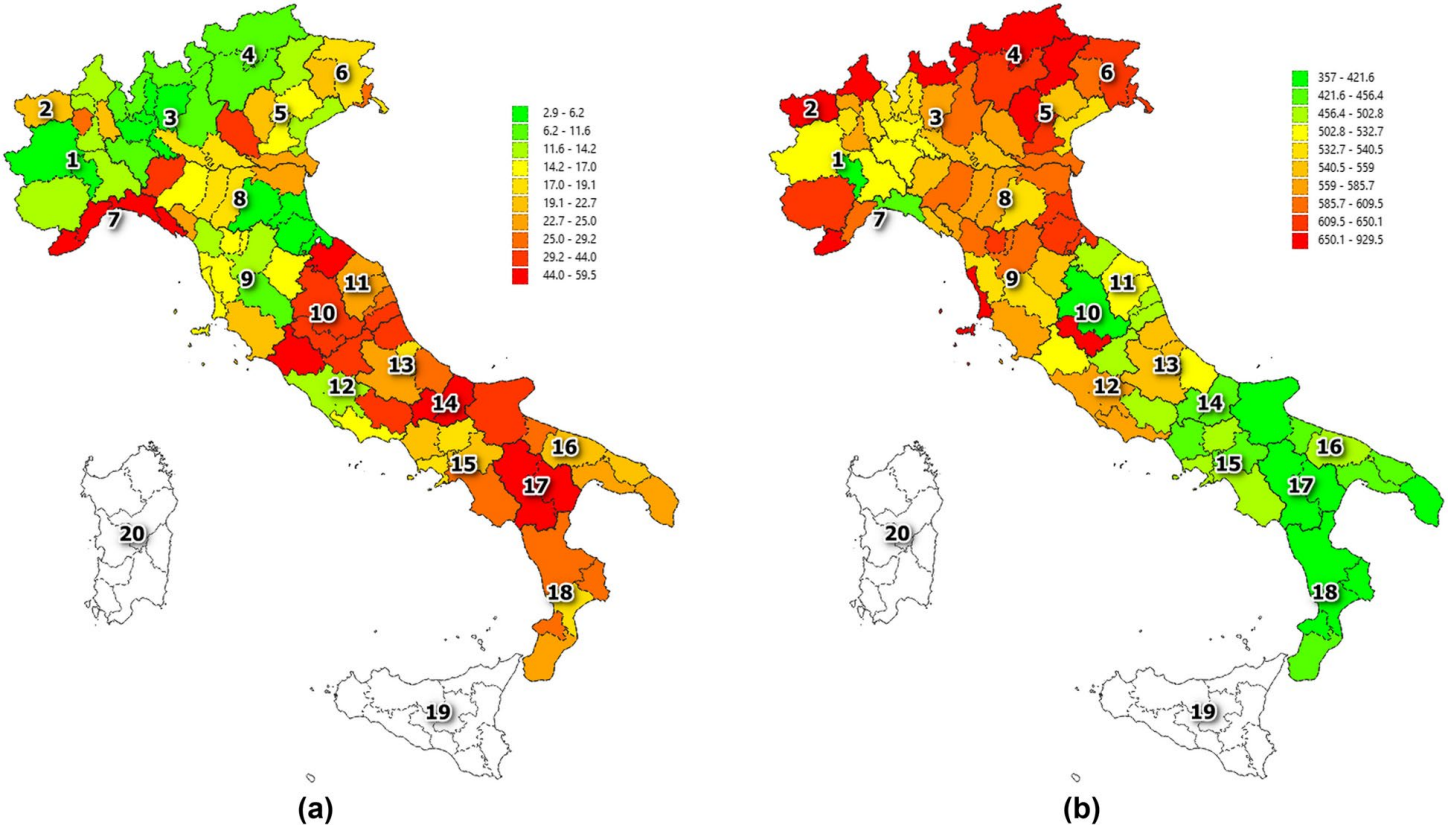
Passive mobility (**a**) and number of patients per population (**b**) at province level. Legend: Reference year: 2022

Figure 4 reports the distribution of passive mobility and the number of patients per population who underwent surgery procedure for hip transplant over the Italian provinces, for the year 2022. As clearly highlighted the south of the country reports a high level of mobility with, however, a low number of hospitalized resident patients. On the contrary, the north of Italy displays a low passive mobility with a high number of patients admitted for hip replacement procedures.

### Econometric model

The summary statistics of the variables included in the econometric models is reported in Table 5 considering the entire sample of 261 provinces/year observations (87 provinces, 3 years).

**Table 5:** Summary statistics of the variables adopted in the econometric analysis

**Variable**	**Average**	**Standard deviation**	**Minimum**	**Maximum**
*Mobility (M)*	0.200	0.131	0.019	0.640
*Intervention (I*^*INTRA*^*)*	3981.6	1482.5	465.9	7359.5
*Intervention (I*^*INTER*^*)*	1207.7	890.2	0.1	4352.0
*Intervention (I*^*G*^*)*	2773.9	1737.1	-3119.5	6593.9
*Intervention (Q*^*INTRA*^*)*	14.15	10.85	0.57	46.41
*Intervention (Q*^*INTER*^*)*	3.75	3.76	0	28.22
*Intervention (Q*^*G*^*)*	10.40	10.77	20.13	38.08
*Income*	18,540.9	3779.0	11,739.9	33,317.3
*Education level*	62.88	7.26	42.70	76.80
*Waiting times*	79.47	33.78	20.79	276.77
*Health expenditure*	2137.00	227.07	1810.90	2822.96
*Specialists*	29.95	3.56	23.76	38.15
*Satisfaction*	46.10	11.21	9.80	77.10

Summary statistics reporting the number of observations, the average and standard deviation as well as the minimum and maximum values

In this study, we performed two analyses: in the first one the econometric model was limited to the two gravity indices (i.e., number of interventions *I*^*G*^ and quality level *Q*^*G*^) to capture to what extent these two accessibility measures influence patient mobility. This model was subsequently extended including the socio-economic and territorial factors reported in Table 2.

Considering the first model, the outlier detection technique using the Cook’s distance led to identify and remove 17 data points (5 in 2020, 5 in 2021 and 7 in 2022) and thus to define an unbalanced panel of 244 samples distributed among 84 provinces in the three-year time span. The result of the regression model is shown in Table 6.

**Table 6:** Results of the panel regression analysis

**Variable**	**Beta coefficient**	**Standard error**	**Significance level**
*Intervention (I*^*G*^*)*	-0.51	0.07	***
*Quality level (Q*^*G*^*)*	-0.42	0.09	***
number of observations (province/year)	244		
number of provinces	84		
*R*^2^	0.43		

Result of the panel analysis reporting for each independent variable, the beta coefficient, standard error and the level of significance with *** for *p* < 0.001

The regression results show that both indicators (interventions and quality level) have a strong negative effect on patient mobility, meaning that the higher is the attraction of intraregional hospitals (compared to the interregional ones), the lower is the portion of patients that require services outside their region of residence.

Table 7 reports the results of the extended econometric model that includes socio-economic and territorial factors (see Table 2). Considering outlier detection, the Cook’s distance led to identify and remove 17 data points (5 in 2020, 4 in 2021 and 8 in 2022) and thus to define an unbalanced panel of 244 samples distributed among 84 provinces in the three-year time span.

**Table 7:** Results of the panel regression analysis

Variable	Beta coefficient	Standard error	Significance level
*Intervention (I*^*G*^*)*	-0.74	0.07	***
*Quality level (Q*^*G*^*)*	-0.23	0.09	**
*Income*	0.09	0.09	
*Education level*	0.18	0.07	**
*Waiting times*	0.19	0.08	**
*Health expenditure*	-0.15	0.04	**
*Specialists*	-0.03	0.04	
*Satisfaction*	-0.06	0.08	
*Position (NUTS level)*	0.31	0.04	***
number of observations (province/year)	244		
number of provinces	84		
R^2^	0.62		

Result of the panel analysis reporting for each independent variable, the beta coefficient, standard error and the level of significance with *** for *p* < 0.001, ** for *p* < 0.01

The regression results of this extended model confirm the significant effect of both the number of interventions and quality level already noted in the previous analysis. Also in this case, this strong correlation has a negative effect on patient mobility, meaning that the higher is the attraction of intraregional hospitals (compared to interregional ones); the lower is the portion of patients that require services in a structure outside their region of residence. Additional variables showing a positive and significant effect are Education level and Waiting times, confirming the results reported in the literature. Considering the former variable, a lower level of education negatively affects the choice of the healthcare facility and the propensity to travel long distances for care [32]. Different studies have highlighted that also Waiting times have a negative and significant impact on hospital demand [76, 77] and this is attributable to two distinct aspects: on the one hand there is the need to carry out the surgical intervention on the basis of the urgency of the clinical case and the patient is willing to choose a structure with a reasonable waiting list. On the other hand, a long waiting list may be considered as a negative indicator considered by the patient to choose a structure with a high-level of quality of care. Health expenditure has been found to show a negative and significant effect on patient mobility, confirming that regions with a high level of investments for the provision of health care services generally report a low level of patient mobility. Finally, a positive and significant effect has been found considering the location of the relevant province. As reported in the methods section we classified each province as north, centre or south on the basis of the NUTS first-level classification (i.e., we set 0 for northern provinces, 1 for central provinces and 2 for southern provinces). The result highlights that there is an increase in passive mobility going from the north of the country and down to the southern provinces. Looking at the significant level of the two dummy variables it emerges that both 2021 and 2022 affected passive mobility. Finally, even if the remaining factors did not show any significant correlation with passive mobility it is interesting to note that they are in line with the findings published on this topic: it is in fact proved that a greater willingness to travel is associated with a higher income [78, 79] as well as with a higher number of specialists [80], while patients’ satisfaction have a positive correlation on patient loyalty and may have a negative effect on patient mobility [81].

## Discussion

The main aim of this study was to capture whether and to what extent the spatial accessibility to quality hospital structures influences on interregional patient mobility in Italy, with a specific focus on hip transplant surgical procedures. Moving from the region of residence to another to access care services, in fact, is an important indicator to evaluate the adequacy of the regional resources and proper healthcare planning [38]. An unevenly distribution of care resources may have an impact in achieving universal health coverage under both supply and demand perspectives. On the one hand, it may expose the population to unequal access to high-quality care with severe clinical and economic consequences. On the other hand, it can contribute to contrast a phenomenon that risks to blow up the regional balance as treating not resident patients implies economic compensation procedures from the patient’s region of residence. Spatial accessibility has been computed using the widely diffused E2SFCA with two main variants: the level of attraction of each hospital is described by quality indicators (i.e., number of interventions and readmissions within 30 days after surgery) as an alternative to traditional structural variables (e.g., number of beds); the level of accessibility is computed considering the gravity model where the attraction due to interregional facilities is subtracted from that of intraregional ones. This approach provided an alternative perspective in determining the level of (in) equity in accessing to qualitative services at territorial level. Looking at the data in depth two main patterns of hospital distribution may be highlighted: in the first one, regions tend to concentrate quality structures in or nearby high populated cities, leaving rural areas not served by this surgery service, confirming the main findings reported in [82] and in our previous analysis [12] regarding critical care. This is the case of Lombardia, Lazio and Campania, regions with the three biggest cities in terms of both area of expansion and resident population. In the second pattern, on the contrary, health systems fairly distributed hospitals over the territory not privileging high density areas and localizing them also at the regional borders. This is mainly found in small regions located in the southern part of the country, such as Molise. In this case, even if these regions showed a low level of intraregional accessibility, they provide an equitable distribution of capacious and quality resources. Another important pattern confirmed by our study is the crucial gap between the northern and the southern part of the country in term of both intra and interregional accessibility components, emphasising the high level of inequity in the accessibility of quality resources. However, exceptions have been found Valle d’Aosta and Liguria two northern regions located at the national borders that are characterized by peripheral, mountain areas with a lack of high-speed road infrastructures. This highlights that the gap between north and south of Italy in terms of spatial accessibility is a mix of factors that may penalize rural municipalities: not only the distribution of high-quality structures that are mainly located in the north of the country but also the presence and modernity of high-speed roads that connect the demand and supply areas in the regional territory.

The second part of the paper explores topic of interregional hospital passive mobility. Also in this case, despite some distinctions, the north–south pattern is confirmed with the highest percentages of patients travelling for hip transplant procedures coming from the south of Italy. The econometric models confirm the high level of dependence between accessibility and passive mobility. Considering the additional variables included in the analysis, it emerged that patients with high level of education are more inclined to travel for clinical purposes and to find out which is the best structure to contact for accessing healthcare services. From a system perspective, two variables are associated with passive mobility: high waiting times and low health expenditure invested by the relevant region. The former may be considered an attractive factor that led patients to choose a specific structure considering it not only as an important quality indicator but also on the basis of the urgency of the clinical case and the necessity of accessing the service as soon as possible. Health expenditure significance confirms that a high level of investments for the provision of health care services in general is closely related with the capacity and quality of services. Finally, the extended model also includes the location of each province within the first-level NUTS classification of the EU, namely north, centre and south. The result of the statistical analysis confirms the main findings reported in the literature [33, 38] and in our previous research [16, 53, 75], highlighting that patients living in the south of Italy prefer to be hospitalized in facilities located outside their region of residence than accessing those located in their residence one. It can be linked with the north–south deprivation gradient, where the majority of deprived municipalities are located in the south of the country [83]. This result is even more interesting considering that the prevalence of patients who underwent to this elective surgery is higher in the northern and central regions than in the south part of the country. This underlines that these regions would be able to compensate for their internal demand confirming that patient’s choice for this elective treatments significantly depends on a combination of both distance from and quality of healthcare resources, confirming the results reported in the literature [37, 84–89]. The reduction of interregional patient mobility, that is considered one of the main objectives of the Italian Ministry of Health as stated in the Health Pact 2019–2021 [34], require to put in place strategies from central and regional governments in order to fill the territorial gap in the supply of an equitable distribution of services not only considering the availability and distribution of services but also their level of attraction in terms of quality [33].

In this complex situation, despite Italy is a decentralized healthcare system, a significant role in reducing inequalities between south and north in order to manage patient mobility may be played by the Ministry of Health. Central government can foster interregional agreements and promote cooperation among regions as well as incentivize and finance new investments in the southern regions [88, 89]. Central and regional governments should consider to put in place some form of mobility regulation and incentives for the hospitals that seek to reduce the quality gap [90]. For instance, as proposed by Balia et al., [29] the introduction of appropriate equalising compensation schemes may neutralize the financial consequences of mobility and, eventually, pledges universalism and equity in healthcare.

## Conclusions

This paper proposed an alternative approach to assess the level of distribution of qualitative hospital resources across Italian regions. Based on the E2SFCA methodology it compares the overall hospital level of attraction coming from intraregional and interregional resources adopting quality (i.e., process and outcome) indicators. This index has been adopted to assess the distribution of structures across Italy and to determine to what extent a patient is encouraged to remain in his/her region of residence to be treated as interregional patient mobility is considered a fundamental phenomenon to analyse equity and for policy planning. The results of the statistical analysis confirmed our hypothesis and are in line with our previous studies [16, 26, 75], underlying that both service capacity and quality level are strictly correlated with mobility and thus confirming that, in Italy, one of the main factors influencing patient’s choice is related not only with socio-economic characteristics of patients or territorial and quality features of hospitals and systems but also with the distance that each patient has to travel to access to care services. The results of this study can contribute to gain a deeper understanding of the allocation of health resources providing input for policy makers on the basis of the principles of service accessibility in order to contain patient mobility. Given that different patters of mobility can be detected, the application of this methodology should be applied to alternative elective surgery procedures as well as to other curative and prevention services, such as primary care, acute care or critical services.

## Data Availability

The dataset supporting the conclusions of this article is available in the Zenodo repository at the following link: https://zenodo.org/doi/10.5281/zenodo.12734087.

## Abbreviations

WHO: World Health Organization
UHC: Universal Health Coverage
SDG: Sustainable Development Goal
IHR: International Health Regulations
2SFCA: Two-Step Floating Catchment Area
E2SFCA: Enhanced Two-Step Floating Catchment Area
PNE: Italian National Outcome Programme
ISTAT: Italian Institute of Statistics
ANOVA: Analysis of Variance
